# Supplementary material for: Temperature-related neonatal deaths attributable to climate change in 29 low- and middle-income countries
Source: Nat Commun. 2024 Jun 29;15:5504. doi: 10.1038/s41467-024-49890-x (PMC11217431; doi:10.1038/s41467-024-49890-x)

Supplementary Information

Temperature-related neonatal deaths attributable to climate change in 29 low- and middle-income countries

Asya Dimitrova[1,2], Anna Dimitrova[3], Matthias Mengel[4], Antonio Gasparrini[5], Hermann Lotze-Campen[1,6], Sabine Gabrysch[1,2,7]

[1] Research Department 2, Potsdam Institute for Climate Impact Research (PIK), Member of the Leibniz Association, Potsdam, Germany

[2]Institute of Public Health, Charité – Universitätsmedizin Berlin, corporate member of Freie Universität Berlin and Humboldt-Universität zu Berlin, Berlin, Germany

[3] Scripps Institution of Oceanography, University of California, San Diego, CA 92037

[4] Research Department 3, Potsdam Institute for Climate Impact Research (PIK), Member of the Leibniz Association, Potsdam, Germany

[5] Environment & Health Modelling (EHM) Lab, Department of Public Health Environments and Society, London School of Hygiene & Tropical Medicine, London, UK

[6] Department of Agricultural Economics, Faculty of Life Sciences, Humboldt University of Berlin, Berlin, Germany

[7] Heidelberg Institute of Global Health, Heidelberg University, Heidelberg, Germany

Supplementary Table 1. Outcome and exposure summary statistics for the 29 study countries, Demographic Health Survey (DHS) data

Region	Country	Survey year	Data period of recorded births	Births	Neonatal deaths	Very early neonatal deaths	Daily mean temperature (°C) *
Europe	**Albania**	2017	2003-2016	8943	30	10	12.6
Sub-Saharan Africa	**Angola**	2015	2001-2016	32636	798	376	22.0
Asia	**Armenia**	2016	2002-2015	4917	42	9	7.3
Asia	**Bangladesh**	2017	2003-2018	26384	875	359	25.8
Sub-Saharan Africa	**Benin**	2017	2003-2018	32775	1073	380	27.9
Sub-Saharan Africa	**Burundi**	2016	2002-2016	34203	865	224	20.8
Sub-Saharan Africa	**Cameroon**	2018	2004-2018	24814	773	253	25.0
Sub-Saharan Africa	**Ethiopia**	2016 & 2019	2002-2019	43174	1602	622	23.3
Sub-Saharan Africa	**Guinea**	2018	2004-2018	20966	670	250	26.2
Carribean	**Haiti**	2016	2002-2017	17954	511	113	25.2
Asia	**India**	2020	2005-2019	716667	17666	6760	24.4
Asia	**Jordan**	2017	2003-2017	31506	292	47	19.5
Sub-Saharan Africa	**Liberia**	2019	2005-2019	16236	606	275	25.6
Sub-Saharan Africa	**Malawi**	2015	2001-2015	48184	1286	573	22.4
Sub-Saharan Africa	**Mali**	2018	2004-2018	24730	863	301	29.0
Asia	**Nepal**	2016	2002-2016	15548	481	241	13.8
Sub-Saharan Africa	**Nigeria**	2018	2004-2018	90221	3168	1318	27.3
Asia	**Pakistan**	2017	2003-2017	34920	1291	325	20.7
Asia	**Philippines**	2017	2003-2017	30152	411	187	26.0
Sub-Saharan Africa	**Rwanda**	2019	2005-2019	21316	463	248	19.8

Sub-Saharan Africa	**Senegal**	2017, 2018 & 2019	2003-2019	66386	1797	302	28.7
Sub-Saharan Africa	**Sierra Leone**	2019	2005-2019	26801	833	311	26.3
Sub-Saharan Africa	**South Africa**	2016	2002-2016	9470	222	136	18.3
Asia	**Tajikistan**	2017	2003-2016	14945	225	37	2.7
Sub-Saharan Africa	**Tanzania**	2015	2001-2016	25890	759	247	22.9
Asia	**Timor-Leste**	2016	2002-2016	21003	363	242	25.07
Sub-Saharan Africa	**Uganda**	2016	2002-2016	40354	1063	447	23.41
Sub-Saharan Africa	**Zambia**	2018	2004-2018	26519	631	270	22.22
Sub-Saharan Africa	**Zimbabwe**	2015	2001-2015	15053	414	164	21.82

*****Estimated as the mean of daily mean temperatures from the three ISIMIP3a reanalysis datasets: GSWP3-W5E5, 20CRV3-ERA5 and 20CRV3-W5E5

Supplementary Table 2. Summary of Minimum Mortality Temperatures across countries for neonatal mortality

Country	Summary of Minimum Mortality Temperature (°C)	
	Average (Minimum, Maximum)	Standard Deviation
**Albania**	13.69 (8.46; 16.82)	2.79
**Angola**	22.92 (18.99; 26.48)	2.28
**Armenia**	9.33 (5.18; 14.55)	1.97
**Bangladesh**	27.24 (21.82; 28.59)	0.9
**Benin**	27.96 (26.84; 29.22)	0.37
**Burundi**	20.7 (18.38; 23.27)	1.33
**Cameroon**	24.92 (20.55; 29.99)	2.17
**Ethiopia**	21.78 (13.75; 30.16)	4
**Guinea**	25.99 (23.92; 28.33)	1.11
**Haiti**	25.61 (22.99; 26.98)	0.86
**India**	25.99 (-1.86; 30.2)	4.1
**Jordan**	20.37 (18.82; 23.84)	0.86
**Liberia**	26.02 (24.48; 26.8)	0.51
**Malawi**	23.26 (18.98; 26.18)	1.52
**Mali**	28.8 (26.56; 31.92)	1.32
**Nepal**	21.5 (-6.1; 28)	5.46
**Nigeria**	27.12 (20.56; 29.64)	1
**Pakistan**	23.13 (-2.81; 30.83)	6.96
**Philippines**	26.45 (19.96; 28.06)	1.4
**Rwanda**	19.3 (16.93; 21.62)	1.35
**Senegal**	28.14 (23.99; 31.01)	1.15
**Sierra Leone**	26.56 (25.12; 27.52)	0.57
**South Africa**	19.16 (10.73; 24.23)	1.95
**Tajikistan**	11.82 (-3.13; 19.78)	5.69
**Tanzania**	23.41 (18.15; 27.25)	2.36
**Timor-Leste**	25.1 (24.55; 26.8)	0.5
**Uganda**	23.02 (16.93; 26.09)	1.72
**Zambia**	22.62 (18.91; 25.99)	0.98
**Zimbabwe**	21.93 (19.08; 26.2)	1.47

*****Calculations based on the estimated MMTs for each DHS cluster in a country and averaged across the three ISIMIP3a reanalysis datasets: GSWP3-W5E5, 20CRV3-ERA5 and 20CRV3-W5E5

Supplementary Table 3. Summary of Minimum Mortality Temperatures across countries for very early neonatal mortality

	**Summary of Minimum Mortality Temperature (°C)**	
**Country**	Average (Minimum, Maximum)	Standard Deviation
**Albania**	10.94 (5.53; 14.27)	2.96
**Angola**	22.37 (18.63; 25.96)	2.22
**Armenia**	4.99 (0.67; 10.66)	2.02
**Bangladesh**	26.01 (20.59; 27.48)	0.97
**Benin**	27.33 (26.34; 28.44)	0.32
**Burundi**	20.4 (18.04; 22.99)	1.34
**Cameroon**	24.51 (20.2; 29.07)	2.07
**Ethiopia**	21.23 (13.37; 29.44)	3.87
**Guinea**	25.56 (23.55; 27.68)	1.1
**Haiti**	25.03 (22.42; 26.28)	0.84
**India**	24.32 (-4.77; 29)	4.42
**Jordan**	17 (15.09; 20.78)	0.93
**Liberia**	25.69 (24.16; 26.44)	0.5
**Malawi**	22.43 (18.24; 25.18)	1.5
**Mali**	27.88 (26.05; 30.39)	1.06
**Nepal**	18.98 (-8.62; 25.85)	5.6
**Nigeria**	26.56 (20.21; 28.69)	0.92
**Pakistan**	19.68 (-5.98; 28.68)	7.1
**Philippines**	26.15 (19.68; 27.75)	1.42
**Rwanda**	19.03 (16.66; 21.32)	1.34
**Senegal**	27.4 (22.6; 29.84)	1.18
**Sierra Leone**	26.22 (24.77; 27.15)	0.56
**South Africa**	17.41 (8.84; 22.49)	2.01
**Tajikistan**	7.84 (-6.7; 15.49)	5.51
**Tanzania**	22.92 (17.56; 26.64)	2.34
**Timor-Leste**	24.83 (24.28; 26.56)	0.49
**Uganda**	22.7 (16.66; 25.68)	1.68
**Zambia**	21.87 (18.24; 25.04)	0.94
**Zimbabwe**	20.64 (17.95; 25.23)	1.5

*****Calculations based on the estimated MMTs for each DHS cluster in a country and averaged across the three ISIMIP3a reanalysis datasets: GSWP3-W5E5, 20CRV3-ERA5 and 20CRV3-W5E5

Supplementary Table 4. AIC model selection for models with 2 lags. Bolded lines indicate models with lowest AIC, which were selected for the analysis.

	Natural spline	B-spline
**Model: Total neonatal mortality**		
1 knot at 10th percentile	118083.9	118090.1
**1 knot at 20th percentile**	**118083.8**	118089.8
1 knot at 30th percentile	118084.3	118090.3
1 knot at 40th percentile	118085.4	118090.3
1 knot at 50th percentile	118087.1	118090.3
1 knot at 60th percentile	118088.8	118090.5
1 knot at 70th percentile	118090.4	118090.8
1 knot at 80th percentile	118091.4	118091.0
1 knot at 90th percentile	118092.0	118090.3
1 knot at 95th percentile	118092.1	118092.0
1 knot at 98th percentile	118092.1	118091.2
2 knots at 25 and 50 th percentile	118085.2	118093.8
2 knots at 50 and 75th percentile	118086.1	118095.2
2 knots at 50 and 90th percentile	118087.0	118094.5
2 knots at 75 and 95th percentile	118092.2	118096.3
3 knots at 10, 50, 75th percentile	118089.8	118099.7
**Model: Very early neonatal mortality**		
**1 knot at 10th percentile**	**29217.38**	29222.16
1 knot at 20th percentile	29217.45	29224.21
1 knot at 30th percentile	29217.85	29224.60
1 knot at 40th percentile	29218.42	29224.75
1 knot at 50th percentile	29219.02	29224.67
1 knot at 60th percentile	29219.67	29224.25
1 knot at 70th percentile	29220.31	29224.15
1 knot at 80th percentile	29220.86	29224.56

1 knot at 90th percentile	29221.11	29224.63
1 knot at 95th percentile	29221.17	29224.71
1 knot at 98th percentile	29221.18	29224.09
2 knots at 25 and 50 th percentile	29220.11	29230.06
2 knots at 50 and 75th percentile	29219.69	29230.30
2 knots at 50 and 90th percentile	29219.51	29230.31
2 knots at 75 and 95th percentile	29220.95	29230.01
3 knots at 10, 50, 75th percentile	29224.92	29231.86

Supplementary Table 5. Heat- and cold-related neonatal mortality fraction (95% confidence interval (CI)) in factual and counterfactual scenario and historical heat- and cold-related neonatal mortality fraction (95% confidence interval (CI)) attributable to climate change by country.

	**Factual scenario**		**Counterfactual scenario**			
**Country**	Heat-related neonatal mortality (% - 95%CI)	Cold-related neonatal mortality (% -95%CI)	Heat-related neonatal mortality (% - 95%CI)	Cold-related neonatal mortality (% -95%CI)	Heat-related neonatal mortality attributable to climate change (% - 95%CI)	Cold-related neonatal mortality attributable to climate change (% - 95%CI)
**Albania**	2.4 (0.4, 4.3)	1.9 (1, 2.7)	2 (0.3, 3.6)	2.2 (1.2, 3.2)	0.5 (0.1, 0.9)	-0.3 (-0.6, -0.2)
**Angola**	1.3 (0.2, 2.6)	3 (1.6, 4.4)	0.7 (0, 1.5)	4.6 (2.5, 6.8)	0.7 (0, 1.4)	-1.5 (-3.4, -0.5)
**Armenia**	0.7 (0, 1.5)	3.7 (2, 5.3)	0.6 (-0.1, 1.2)	4.2 (2.3, 6.1)	0.2 (0, 0.4)	-0.5 (-1.1, -0.2)
**Bangladesh**	1.3 (0.2, 2.3)	3.3 (1.8, 4.8)	0.8 (0, 1.7)	3.7 (2, 5.4)	0.4 (0, 0.8)	-0.4 (-0.8, -0.1)
**Benin**	1.4 (0.2, 2.6)	2.8 (1.5, 4.1)	0.6 (0.1, 1.2)	5.8 (3.2, 8.6)	0.8 (0, 1.5)	-3 (-5, -1.2)
**Burundi**	1.2 (0.2, 2.2)	3 (1.6, 4.4)	0.5 (0, 0.9)	5.8 (3.3, 8.3)	0.7 (0, 1.4)	-2.8 (-4.6, -1.4)
**Cameroon**	1.7 (0.3, 3)	2.3 (1.2, 3.4)	0.9 (0.1, 1.9)	5.8 (2.9, 9.1)	0.8 (0, 1.8)	-3.5 (-6.3, -1)
**Ethiopia**	1.4 (0.2, 2.5)	2.8 (1.5, 4.1)	0.5 (0, 1.1)	6.1 (3.4, 8.8)	0.9 (0, 1.7)	-3.2 (-5.2, -1.5)
**Guinea**	1.8 (0.3, 3.2)	2.7 (1.4, 3.9)	1 (0.1, 2.1)	5.8 (3, 8.9)	0.8 (0, 1.7)	-3.1 (-5.5, -0.9)
**Haiti**	1.4 (0.2, 2.6)	2.9 (1.5, 4.2)	0.3 (0, 0.9)	5.4 (2.9, 8.4)	1.1 (0, 2.3)	-2.5 (-4.9, -1)
**India**	1.5 (0.2, 2.7)	2.8 (1.5, 4)	1.2 (0.2, 2.2)	3.2 (1.6, 4.8)	0.3 (0, 0.6)	-0.4 (-1.1, 0)
**Jordan**	1.3 (0.2, 2.4)	3.3 (1.8, 4.7)	0.8 (0, 1.7)	3.9 (2.1, 5.8)	0.5 (0.1, 1.2)	-0.6 (-1.4, -0.2)
**Liberia**	1.5 (0.3, 2.6)	2.9 (1.5, 4.2)	0.6 (0.1, 1.6)	6.4 (3.2, 10.1)	0.9 (0.1, 2)	-3.5 (-6.6, -1)
**Malawi**	1.3 (0.2, 2.5)	2.8 (1.5, 4)	0.9 (0.1, 1.8)	3.8 (2.1, 5.4)	0.4 (0, 0.8)	-1 (-1.5, -0.5)
**Mali**	1.6 (0.2, 2.9)	3 (1.7, 4.4)	0.9 (0.1, 1.7)	5.1 (2.9, 7.4)	0.7 (0.1, 1.3)	-2 (-3.4, -0.9)
**Nepal**	1.4 (0.2, 2.6)	2.7 (1.4, 3.9)	1 (0.1, 1.9)	3 (1.5, 4.6)	0.4 (0.1, 0.8)	-0.3 (-1.1, 0)
**Nigeria**	1.6 (0.3, 2.9)	2.6 (1.4, 3.8)	0.9 (0.1, 1.8)	4.8 (2.6, 7.3)	0.6 (0, 1.3)	-2.2 (-3.9, -0.8)
**Pakistan**	1.4 (0.2, 2.5)	3 (1.6, 4.3)	1.1 (0.1, 2.1)	3.5 (1.9, 5)	0.3 (0, 0.7)	-0.5 (-1, -0.2)
**Philippines**	1.3 (0.2, 2.4)	3 (1.5, 4.4)	0.3 (0, 0.6)	7.6 (4.4, 10.9)	1 (0, 2)	-4.6 (-7, -2.4)
**Rwanda**	1.2	2.8	0.4	6.6	0.9	-3.8

	(0.1, 2.3)	(1.3, 4.4)	(0, 0.8)	(3.8, 9.4)	(-0.1, 1.7)	(-6, -2)
**Senegal**	1.4<br>(0.2, 2.6)	2.9<br>(1.5, 4.1)	0.7<br>(0.1, 1.4)	5.2<br>(2.9, 7.7)	0.7<br>(0, 1.4)	-2.4<br>(-4, -1)
**Sierra Leone**	1.9<br>(0.3, 3.3)	2.3<br>(1.1, 3.4)	1<br>(0.1, 2.5)	5.1<br>(2.5, 8.4)	0.9<br>(0, 2.2)	-2.8<br>(-5.6, -0.7)
**South Africa**	1.4<br>(0.1, 2.6)	3<br>(1.6, 4.2)	0.8<br>(0, 1.5)	4.2<br>(2.3, 6)	0.6<br>(0, 1.2)	-1.2<br>(-2, -0.6)
**Tajikistan**	1.6<br>(0.3, 3)	2.2<br>(1.2, 3.3)	1.3<br>(0.2, 2.4)	2.6<br>(1.3, 3.8)	0.3<br>(0.1, 0.6)	-0.3<br>(-0.8, -0.1)
**Tanzania**	1.1<br>(0.1, 2.1)	3.3<br>(1.8, 4.8)	0.5<br>(0, 1)	5.7<br>(3.3, 8)	0.6<br>(0, 1.2)	-2.4<br>(-3.5, -1.3)
**Timor-Leste**	1.2<br>(0.1, 2.2)	3.2<br>(1.7, 4.6)	0.4<br>(0, 1)	5.3<br>(3, 7.6)	0.8<br>(0, 1.5)	-2.1<br>(-3.7, -1)
**Uganda**	1.5<br>(0.3, 2.8)	2.7<br>(1.4, 3.9)	0.6<br>(0.1, 1.2)	7.1<br>(4.2, 10)	0.9<br>(0, 1.8)	-4.5<br>(-6.4, -2.3)
**Zambia**	1.4<br>(0.2, 2.5)	3<br>(1.6, 4.4)	1<br>(0.1, 2)	3.9<br>(2, 5.7)	0.3<br>(0, 0.9)	-0.9<br>(-1.7, -0.4)
**Zimbabwe**	1.4<br>(0.1, 2.6)	2.6<br>(1.4, 3.8)	0.9<br>(0.1, 1.8)	3.5<br>(1.9, 5)	0.5<br>(0, 0.9)	-0.9<br>(-1.3, -0.4)

Supplementary Table 6. Heat-related neonatal deaths attributable to climate change and cold-related neonatal deaths averted by climate change by country, expressed as rates, total numbers and as a share of the total heat- and cold-related burdens and including 95% Uncertainty Intervals (UI)

Country	Heat-related neonatal deaths attributable to climate change			Cold-related neonatal deaths averted by climate change		
	Rate (per 100,000 births)	Total number	Share of all heat-related burden (%) *	Rate (per 100,000 births)	Total number	Share of all cold-related burden (%) **
**Albania**	4<br>(1, 8)	27<br>(4, 56)	19	-3<br>(-5, -1)	-20<br>(-35, -9)	15
**Angola**	24<br>(0, 50)	4825<br>(68, 9999)	50	-55<br>(-123, -19)	-10911<br>(-24454, -3769)	33
**Armenia**	2<br>(0, 4)	12<br>(1, 31)	22	-5<br>(-11, -2)	-42<br>(-82, -17)	13
**Bangladesh**	13<br>(1, 24)	8064<br>(873, 15281)	34	-12<br>(-26, -3)	-7347<br>(-16117, -2192)	10
**Benin**	27<br>(1, 52)	1976<br>(69, 3742)	56	-101<br>(-170, -41)	-7243<br>(-12197, -2976)	52
**Burundi**	21<br>(0, 39)	1516<br>(23, 2909)	62	-79<br>(-128, -38)	-5808<br>(-9442, -2823)	49
**Cameroon**	26<br>(1, 55)	3826<br>(121, 8206)	49	-108<br>(-196, -32)	-16173<br>(-29382, -4808)	60
**Ethiopia**	33<br>(1, 64)	20537<br>(906, 40511)	64	-121<br>(-195, -56)	-76016<br>(-122510, -35172)	53
**Guinea**	28<br>(2, 61)	2111<br>(138, 4679)	44	-111<br>(-201, -34)	-8484<br>(-15340, -2600)	53
**Haiti**	31<br>(1, 65)	1596<br>(52, 3328)	79	-70<br>(-137, -27)	-3559<br>(-6978, -1386)	46
**India**	9<br>(0, 18)	47046<br>(2433, 90005)	20	-12<br>(-36, -1)	-58805<br>(-180473, -5710)	11
**Jordan**	6<br>(1, 14)	214<br>(26, 530)	38	-7<br>(-17, -2)	-265<br>(-625, -87)	16
**Liberia**	32<br>(2, 71)	896<br>(50, 2018)	61	-124<br>(-231, -33)	-3513<br>(-6527, -942)	55
**Malawi**	11<br>(0, 21)	1234<br>(24, 2359)	31	-26<br>(-40, -12)	-2937<br>(-4487, -1391)	26
**Mali**	28<br>(2, 52)	3821<br>(292, 7117)	44	-81<br>(-136, -35)	-11059<br>(-18489, -4707)	40
**Nepal**	12<br>(2, 23)	1445<br>(219, 2759)	30	-9<br>(-31, 0)	-1123<br>(-3783, -47)	11
**Nigeria**	25<br>(2, 51)	31701<br>(2015, 64325)	40	-86<br>(-153, -30)	-109236<br>(-193958, -38248)	46
**Pakistan**	14<br>(0, 34)	16632<br>(250, 39843)	21	-23<br>(-49, -8)	-26443<br>(-56785, -9542)	13
**Philippines**	16<br>(0, 30)	7043<br>(-103, 13711)	79	-69<br>(-104, -35)	-31221<br>(-47309, -15886)	61

**Rwanda**	22<br>(-2, 44)	1484<br>(-139, 3017)	70	-96<br>(-152, -50)	-6619<br>(-10484, -3430)	57
**Senegal**	20<br>(1, 40)	1772<br>(73, 3564)	50	-65<br>(-110, -29)	-5810<br>(-9798, -2576)	45
**Sierra Leone**	35<br>(2, 87)	1623<br>(74, 4046)	47	-113<br>(-222, -27)	-5255<br>(-10352, -1262)	55
**South Africa**	8<br>(0, 15)	1630<br>(24, 3289)	43	-16<br>(-25, -7)	-3375<br>(-5367, -1590)	29
**Tajikistan**	7<br>(1, 12)	296<br>(46, 525)	21	-6<br>(-15, -2)	-273<br>(-650, -67)	12
**Tanzania**	16<br>(0, 30)	5405<br>(54, 10387)	56	-60<br>(-89, -32)	-20874<br>(-30904, -11084)	42
**Timor-Leste**	22<br>(0, 43)	143<br>(-1, 283)	65	-61<br>(-105, -28)	-400<br>(-684, -185)	40
**Uganda**	23<br>(1, 45)	6038<br>(174, 11839)	59	-113<br>(-162, -59)	-29784<br>(-42859, -15537)	63
**Zambia**	9<br>(0, 26)	995<br>(38, 2750)	24	-24<br>(-46, -10)	-2536<br>(-4987, -1043)	22
**Zimbabwe**	14<br>(0, 27)	1225<br>(2, 2407)	33	-26<br>(-39, -12)	-2253<br>(-3461, -1020)	24

*Calculated with respect to heat-related neonatal deaths in the factual scenario.

**Calculated with respect to cold-related neonatal deaths in the counterfactual scenario.

Supplementary Table 7. Heat- and cold-related very early neonatal mortality fraction (95% confidence interval (CI)) in factual and counterfactual scenario and historical heat- and cold-related neonatal mortality fraction (95% confidence interval (CI)) attributable to climate change by country.

	Factual scenario		Counterfactual scenario			
**Country**	Heat-related very early neonatal mortality (% - 95%CI)	Cold-related very early neonatal mortality (% -95%CI)	Heat-related very early neonatal mortality (% - 95%CI)	Cold-related very early neonatal mortality (% -95%CI)	Heat-related very early neonatal mortality attributable to climate change (% - 95%CI)	Heat-related very early neonatal mortality attributable to climate change (% - 95%CI)
**Albania**	7.7 (3, 12)	0.3 (-0.2, 0.7)	6.7 (2.6, 10.5)	0.4 (-0.1, 1)	1 (0.2, 1.8)	-0.2 (-0.3, 0)
**Angola**	3.8 (1.4, 6.1)	2.3 (0.3, 4.2)	2 (0.5, 3.8)	3.4 (0.6, 6.2)	1.9 (0.6, 3.4)	-1.1 (-3, 0)
**Armenia**	0.2 (-0.1, 0.5)	3.5 (0.3, 6.5)	0.2 (0, 0.4)	4.1 (0.4, 7.6)	0 (-0.1, 0.1)	-0.6 (-1.6, 0)
**Bangladesh**	3.9 (1.5, 6.2)	2.1 (0.2, 3.9)	2.9 (0.8, 4.8)	2.4 (0.2, 4.5)	1.1 (0.4, 1.8)	-0.3 (-0.9, 0)
**Benin**	3.9 (1.5, 6.1)	2 (0.2, 3.7)	1.8 (0.6, 3)	4.5 (0.9, 8.1)	2.1 (0.6, 3.4)	-2.5 (-5.1, -0.2)
**Burundi**	4.2 (1.7, 6.4)	1.8 (0.1, 3.3)	1.7 (0.5, 2.8)	3.9 (0.5, 7)	2.5 (0.9, 4)	-2.1 (-4.3, 0.1)
**Cameroon**	4.7 (1.9, 7.2)	1.6 (0.2, 3)	2.3 (0.7, 4.5)	4.3 (0.8, 8.4)	2.4 (0.7, 4.4)	-2.7 (-6.3, 0)
**Ethiopia**	3.9 (1.5, 6.1)	1.8 (0.1, 3.5)	1.6 (0.5, 2.9)	4.4 (0.6, 8.1)	2.3 (0.7, 4)	-2.6 (-5.3, -0.1)
**Guinea**	5.2 (1.9, 8.1)	1.6 (0, 3.2)	3.1 (0.8, 5.9)	4.1 (0.7, 7.9)	2.1 (0.6, 4.1)	-2.4 (-5.5, -0.2)
**Haiti**	4.3 (1.6, 6.8)	2 (0.2, 3.8)	1.4 (0.2, 3.1)	3.8 (0.5, 7.4)	2.9 (0.8, 5.5)	-1.7 (-4.4, 0.1)
**India**	4.2 (1.6, 6.6)	1.9 (0.2, 3.5)	3.5 (1.3, 5.6)	2.1 (0.2, 4.1)	0.7 (0.2, 1.2)	-0.3 (-1.1, 0)
**Jordan**	3.9 (1.5, 6.1)	2.2 (0.2, 4)	2.6 (0.8, 4.5)	2.7 (0.3, 4.9)	1.4 (0.4, 2.8)	-0.5 (-1.3, 0)
**Liberia**	4.4 (1.8, 6.8)	1.8 (0, 3.5)	1.8 (0.4, 4.4)	4.6 (0.7, 9.1)	2.6 (0.7, 5.2)	-2.8 (-6.5, -0.2)
**Malawi**	3.7 (1.3, 5.9)	2 (0.2, 3.8)	2.7 (0.8, 4.4)	2.9 (0.3, 5.3)	1 (0.4, 1.6)	-0.9 (-1.7, 0)
**Mali**	4.2 (1.6, 6.7)	2.2 (0.3, 4)	2.6 (0.9, 4.3)	3.8 (0.6, 6.9)	1.6 (0.5, 2.6)	-1.6 (-3.5, 0)
**Nepal**	3.7 (1.4, 5.8)	1.8 (0.1, 3.3)	2.9 (1, 4.6)	2.1 (0.2, 4.1)	0.8 (0.3, 1.4)	-0.3 (-1.3, 0)
**Nigeria**	4.2 (1.6, 6.6)	1.8 (0.1, 3.5)	2.5 (0.8, 4.3)	3.8 (0.6, 7.2)	1.7 (0.5, 3.1)	-2 (-4.5, -0.1)
**Pakistan**	3.8 (1.4, 6)	1.7 (0.1, 3.3)	3.2 (1, 5.4)	2.1 (0.2, 4)	0.6 (0, 1.3)	-0.4 (-1.1, 0)
**Philippines**	4.4	2.1	1.3	5.2	3.2	-3.1

	(1.7, 7)	(0.3, 3.8)	(0.3, 2.2)	(0.9, 9.3)	(1, 5.2)	(-6.3, 0.1)
**Rwanda**	3.7<br>(1.3, 6)	2.2<br>(0.2, 4.3)	1.3<br>(0.3, 2.3)	5.1<br>(0.9, 9)	2.4<br>(0.6, 4.1)	-2.9<br>(-5.8, 0)
**Senegal**	4.2<br>(1.5, 6.8)	1.6<br>(0.2, 3)	2.3<br>(0.7, 4)	3.4<br>(0.6, 6.3)	1.9<br>(0.5, 3.5)	-1.8<br>(-3.9, -0.1)
**Sierra Leone**	5<br>(2, 7.7)	1.6<br>(0.1, 3)	2.8<br>(0.7, 6.1)	3.9<br>(0.6, 8.2)	2.1<br>(0.4, 4.6)	-2.4<br>(-6.1, -0.1)
**South Africa**	3.9<br>(1.5, 6.1)	2.6<br>(0.4, 4.7)	2.6<br>(0.9, 4.1)	3.6<br>(0.6, 6.5)	1.3<br>(0.4, 2.3)	-1<br>(-2.1, 0)
**Tajikistan**	5.1<br>(2.1, 7.8)	2<br>(0.2, 3.8)	4.3<br>(1.7, 6.8)	2.3<br>(0.2, 4.3)	0.8<br>(0.3, 1.2)	-0.2<br>(-0.8, 0)
**Tanzania**	3<br>(1, 4.9)	2.3<br>(0.1, 4.4)	1.5<br>(0.3, 2.6)	4.2<br>(0.6, 7.5)	1.5<br>(0.4, 2.6)	-1.9<br>(-3.5, -0.1)
**Timor-Leste**	3.5<br>(1.2, 5.8)	2.3<br>(0.3, 4.2)	1.4<br>(0.3, 2.7)	3.9<br>(0.6, 7)	2.1<br>(0.6, 3.5)	-1.6<br>(-3.3, 0)
**Uganda**	4.3<br>(1.7, 6.8)	1.7<br>(0.1, 3.2)	1.9<br>(0.7, 3.1)	5.4<br>(1, 9.5)	2.4<br>(0.7, 4.2)	-3.7<br>(-6.8, -0.2)
**Zambia**	3.8<br>(1.4, 6.1)	2.1<br>(0.2, 3.9)	3<br>(1, 5.1)	2.8<br>(0.4, 5.1)	0.9<br>(0.2, 2.1)	-0.7<br>(-1.6, 0)
**Zimbabwe**	4.2<br>(1.4, 6.8)	1.3<br>(0, 2.6)	3<br>(0.9, 5.1)	2<br>(0.1, 3.7)	1.2<br>(0.3, 2)	-0.6<br>(-1.2, 0)

Supplementary Table 8. Heat-related and cold-related very early neonatal deaths attributable to climate change and cold-related very early neonatal deaths averted by climate change by country, expressed as rates, total numbers and share of total heat- and cold-related burdens and including 95% Uncertainty Intervals (UI)

Country	Heat-related very early neonatal deaths attributable to climate change			Cold-related very early neonatal deaths averted by climate change		
	**Rate (per 100,000 births)**	**Total number**	**Share of all heat-related burden (%)**	**Rate (per 100,000 births)**	**Total number**	**Share of all cold-related burden (%)**
**Albania**	3 (1, 5)	18 (4, 33)	13	0 (-1, 0)	-3 (-6, 0)	35
**Angola**	33 (10, 60)	6524 (1923, 11925)	49	-19 (-53, 0)	-3828 (-10451, 95)	32
**Armenia**	0 (0, 0)	0 (-1, 2)	8	-2 (-4, 0)	-12 (-34, 0)	14
**Bangladesh**	13 (5, 22)	8268 (3246, 14026)	27	-4 (-11, 0)	-2485 (-6685, 65)	13
**Benin**	26 (8, 42)	1853 (565, 3009)	54	-31 (-63, -2)	-2203 (-4549, -152)	56
**Burundi**	17 (6, 28)	1284 (445, 2058)	60	-15 (-30, 1)	-1078 (-2236, 59)	54
**Cameroon**	26 (8, 48)	3833 (1143, 7135)	51	-29 (-68, 0)	-4385 (-10214, -72)	63
**Ethiopia**	33 (10, 57)	20859 (6424, 36071)	60	-37 (-76, -1)	-23135 (-47690, -525)	59
**Guinea**	29 (8, 57)	2241 (642, 4357)	41	-34 (-77, -3)	-2593 (-5888, -238)	60
**Haiti**	21 (6, 40)	1066 (300, 2038)	67	-13 (-32, 1)	-648 (-1642, 26)	46
**India**	9 (3, 15)	44044 (13832, 73373)	17	-3 (-14, 0)	-17390 (-68061, 1671)	13
**Jordan**	3 (1, 7)	120 (39, 252)	35	-1 (-3, 0)	-43 (-117, -2)	18
**Liberia**	47 (13, 94)	1340 (364, 2649)	60	-51 (-118, -3)	-1438 (-3343, -96)	61
**Malawi**	12 (4, 19)	1316 (466, 2078)	28	-10 (-19, 0)	-1118 (-2152, -29)	30
**Mali**	23 (7, 37)	3078 (908, 5016)	38	-23 (-49, 0)	-3069 (-6627, -1)	42
**Nepal**	11 (3, 19)	1382 (428, 2350)	22	-4 (-17, 0)	-538 (-2121, 31)	15
**Nigeria**	27 (8, 48)	34071 (10083, 60832)	40	-31 (-70, -2)	-39647 (-88858, -1939)	52
**Pakistan**	8 (0, 17)	8912 (-9, 19705)	16	-5 (-14, 0)	-5684 (-15673, -304)	18

**Philippines**	18<br>(5, 29)	8126<br>(2486, 13184)	72	-18<br>(-35, 0)	-7966<br>(-15967, 153)	60
**Rwanda**	32<br>(8, 55)	2204<br>(562, 3776)	65	-38<br>(-76, 0)	-2647<br>(-5263, -22)	57
**Senegal**	8<br>(2, 15)	723<br>(190, 1319)	45	-8<br>(-17, 0)	-683<br>(-1480, -31)	53
**Sierra Leone**	33<br>(5, 70)	1520<br>(255, 3256)	43	-36<br>(-92, -1)	-1683<br>(-4306, -66)	60
**South Africa**	11<br>(3, 18)	2296<br>(621, 3959)	34	-8<br>(-17, 0)	-1763<br>(-3604, -39)	28
**Tajikistan**	2<br>(1, 4)	105<br>(47, 164)	15	-1<br>(-3, 0)	-33<br>(-114, 0)	10
**Tanzania**	13<br>(4, 22)	4498<br>(1307, 7530)	51	-16<br>(-29, -1)	-5451<br>(-10194, -317)	45
**Timor-Leste**	39<br>(10, 65)	254<br>(68, 422)	60	-30<br>(-61, 0)	-194<br>(-395, 1)	41
**Uganda**	25<br>(7, 43)	6635<br>(1888, 11348)	56	-38<br>(-70, -2)	-10103<br>(-18399, -625)	68
**Zambia**	10<br>(3, 24)	1105<br>(284, 2620)	23	-8<br>(-19, 0)	-874<br>(-2027, -45)	25
**Zimbabwe**	13<br>(4, 23)	1160<br>(325, 1980)	28	-7<br>(-14, 0)	-628<br>(-1241, 25)	32

*Calculated with respect to very early neonatal heat-related deaths in the factual scenario.

**Calculated with respect to cold-related very early neonatal deaths in the counterfactual scenario.

Supplementary Table 9 Summary of total neonatal deaths and very early neonatal deaths for the 29 locations for the period 2001-2019, UNICEF*

Region	Country	Data period	Births	Neonatal deaths	Very early neonatal deaths
**Europe**	Albania	2001-2019	706,962	5,970	1,812
**Sub-Saharan Africa**	Angola	2001-2019	19,876,855	722,089	346,156
**Asia**	Armenia	2001-2019	764,025	7,689	2,075
**Asia**	Bangladesh	2001-2019	62,771,163	1,899,494	784,766
**Sub-Saharan Africa**	Benin	2001-2019	7,189,971	242,006	88,335
**Sub-Saharan Africa**	Burundi	2001-2019	7,385,509	204,773	51,441
**Sub-Saharan Africa**	Cameroon	2001-2019	14,992,573	462,927	161,198
**Sub-Saharan Africa**	Ethiopia	2001-2019	62,897,178	2,354,926	895,125
**Sub-Saharan Africa**	Guinea	2001-2019	7,644,309	276,540	107,141
**Carribean**	Haiti	2001-2019	5,092,004	143,215	37,112
**Asia**	India	2001-2019	500,810,268	16,197,943	6,162,751
**Asia**	Jordan	2001-2019	3,707,282	43,637	8,868
**Sub-Saharan Africa**	Liberia	2001-2019	2,829,920	98,995	51,152
**Sub-Saharan Africa**	Malawi	2001-2019	11,201,088	295,195	128,578
**Sub-Saharan Africa**	Mali	2001-2019	13,633,838	544,775	190,035
**Asia**	Nepal	2001-2019	12,248,923	332,788	168,981
**Sub-Saharan Africa**	Nigeria	2001-2019	126,977,974	4,933,373	1,994,100
**Asia**	Pakistan	2001-2019	115,709,872	5,663,523	1,483,550
**Asia**	Philippines	2001-2019	45,306,761	674,853	254,218
**Sub-Saharan Africa**	Rwanda	2001-2019	6,889,961	173,673	91,025
**Sub-Saharan Africa**	Senegal	2001-2019	8,879,802	246,774	37,917
**Sub-Saharan Africa**	Sierra Leone	2001-2019	4,670,723	185,246	71,061
**Sub-Saharan Africa**	South Africa	2001-2019	21,484,841	274,676	174,347
**Asia**	Tajikistan	2001-2019	4,403,244	86,232	13,894
**Sub-Saharan Africa**	Tanzania	2001-2019	34,788,636	878,558	292,267
**Asia**	Timor-Leste	2001-2019	651,775	18,648	12,006
**Sub-Saharan Africa**	Uganda	2001-2019	26,442,665	665,732	272,191
**Sub-Saharan Africa**	Zambia	2001-2019	10,731,353	296,582	125,318
**Sub-Saharan Africa**	Zimbabwe	2001-2019	8,790,554	264,256	99,770

*Source: UNICEF Data Warehouse. https://data.unicef.org/dv_index/

Supplementary Fig. 1: Time series plots of mean daily temperature for factual and counterfactual scenarios in the 29 study countries, in the time period 1900-2019. Mean values computed across the three reanalysis datasets. The dashed vertical line indicates the start of the study period.

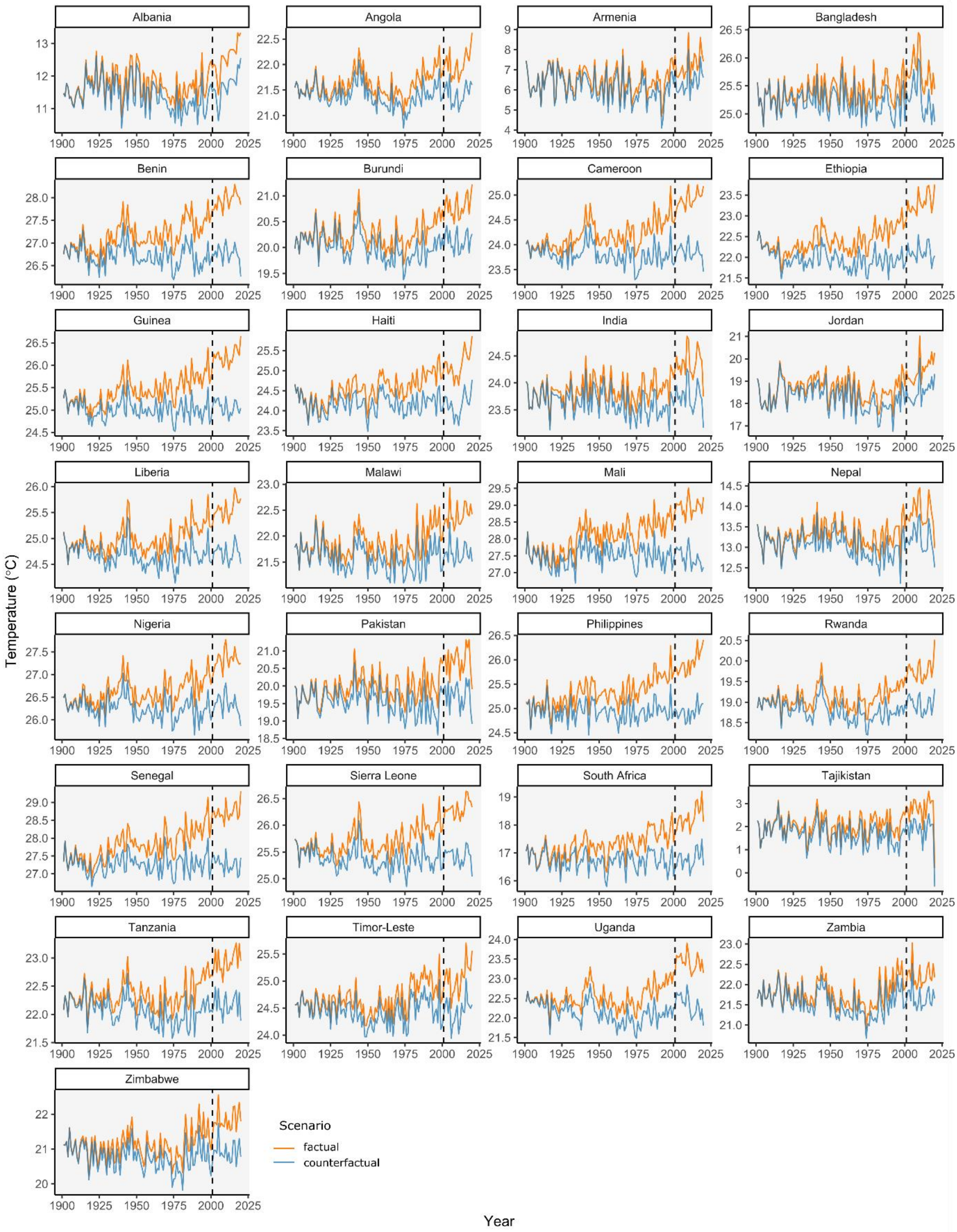

Supplementary Fig. 2: Minimum Mortality Temperatures (MMT) (°C) across DHS clusters. a, neonatal mortality and **b,** very early neonatal mortality. Estimated as the average across the three ISIMIP3a reanalysis datasets: GSWP3-W5E5, 20CRV3-ERA5 and 20CRV3-W5E5.

a

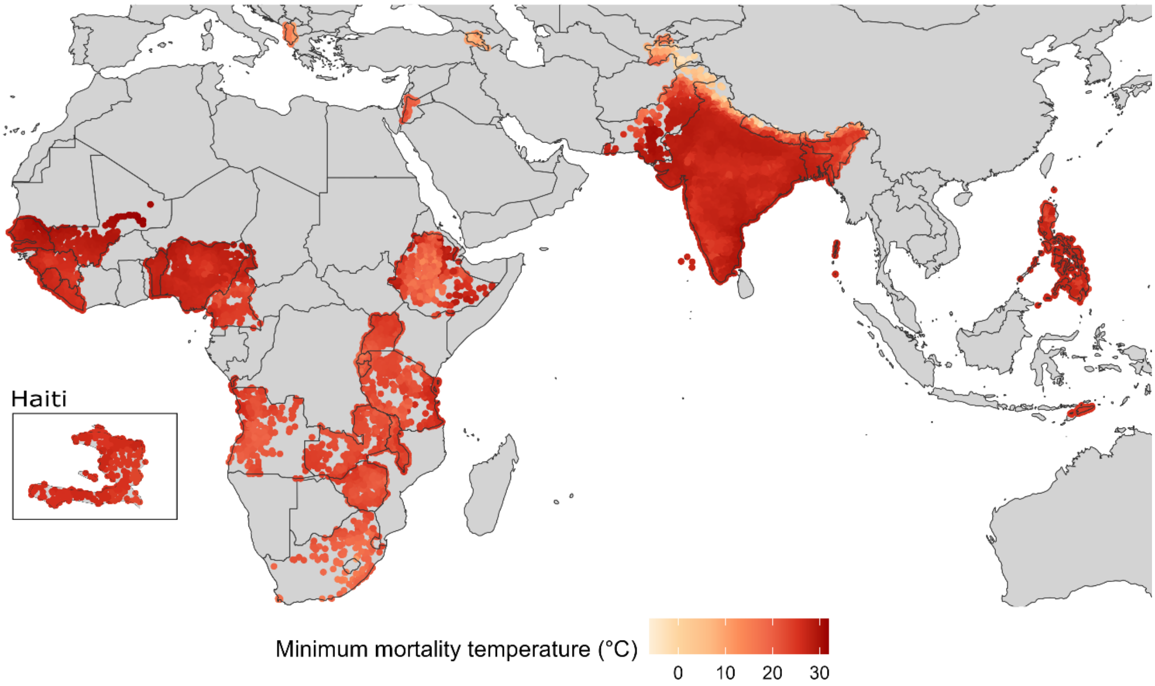

b

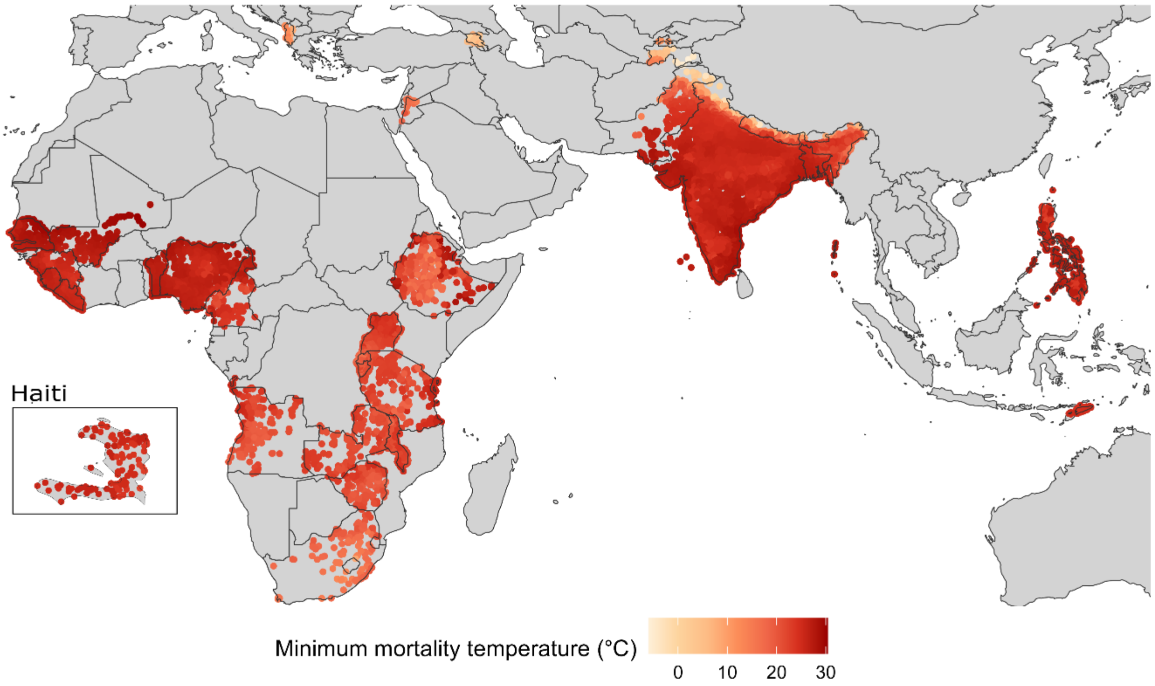

Supplementary Fig. 3: Annual mean temperature and minimum mortality temperature in the 29 countries. **a**, Annual mean temperature and minimum neonatal mortality temperature (°C). **b**, Average latitude and minimum neonatal mortality temperature (°C) **c**, Annual mean temperature and minimum very early neonatal mortality temperature (°C). **d**, Average latitude and minimum very early neonatal mortality temperature (°C)

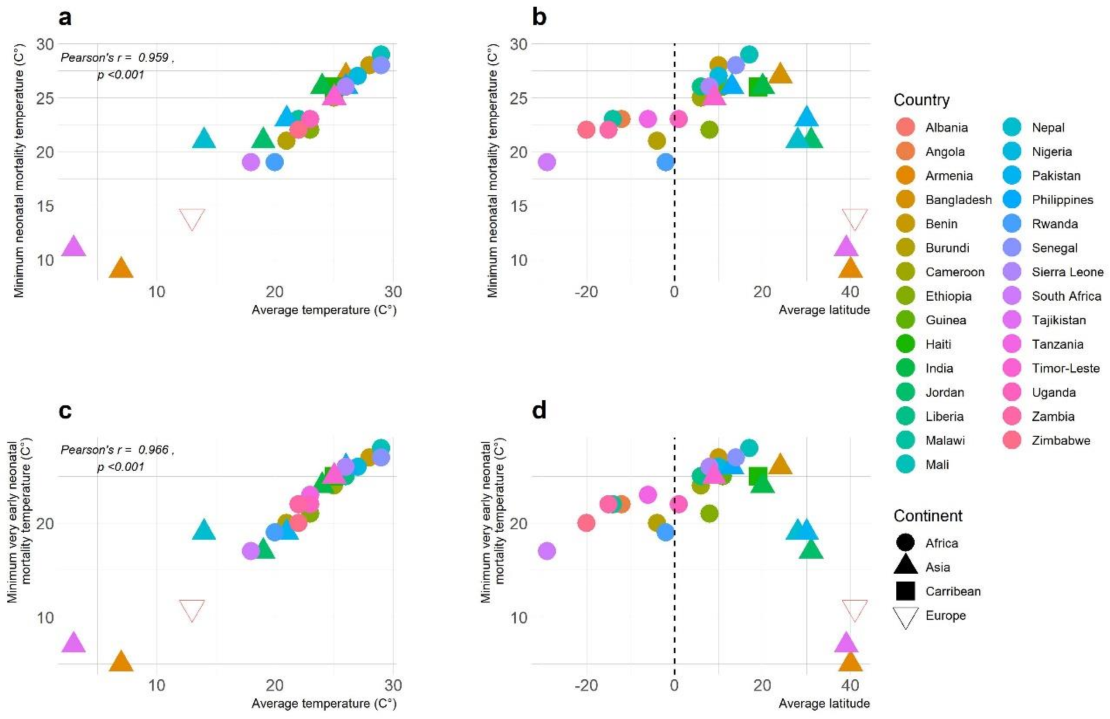

Supplementary Fig. 4: Sensitivity analysis of overall cumulative temperature–neonatal mortality association based on recall period of the mother with temperature dataset gswp3-w5e5

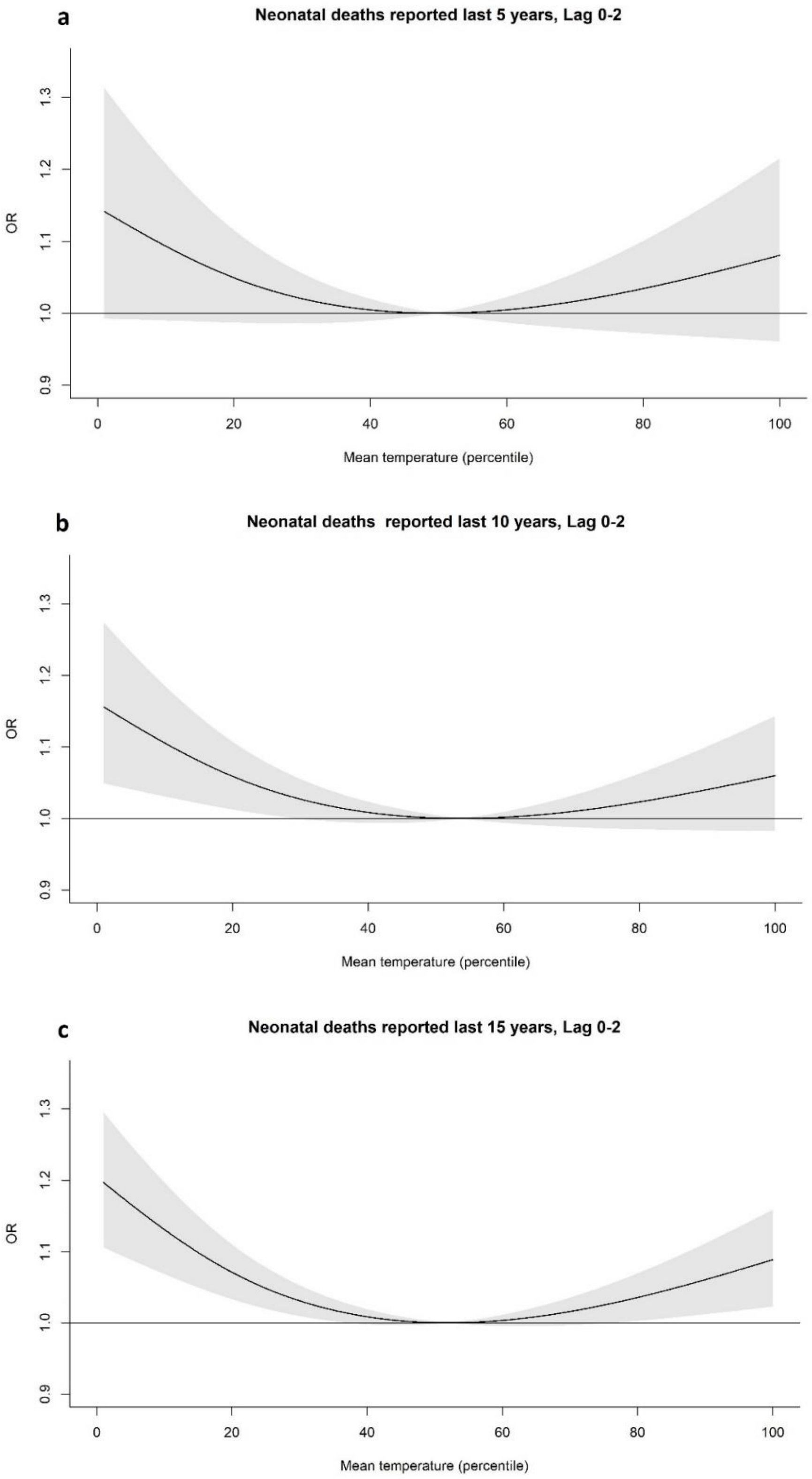

Supplementary Fig. 5: Sensitivity analysis of the overall cumulative temperature–neonatal mortality association based on knots number and placement with temperature dataset gswp3-w5e5

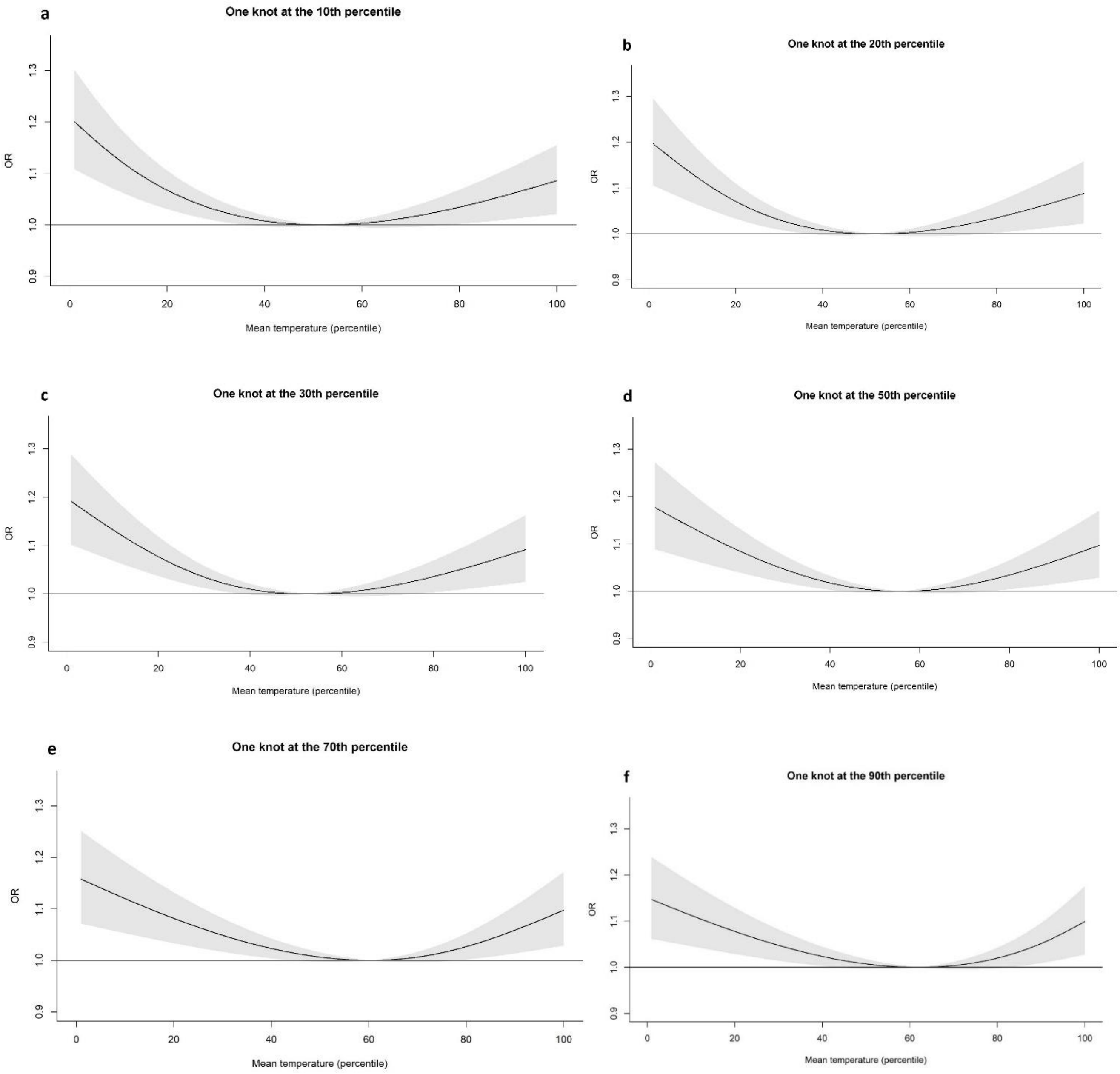

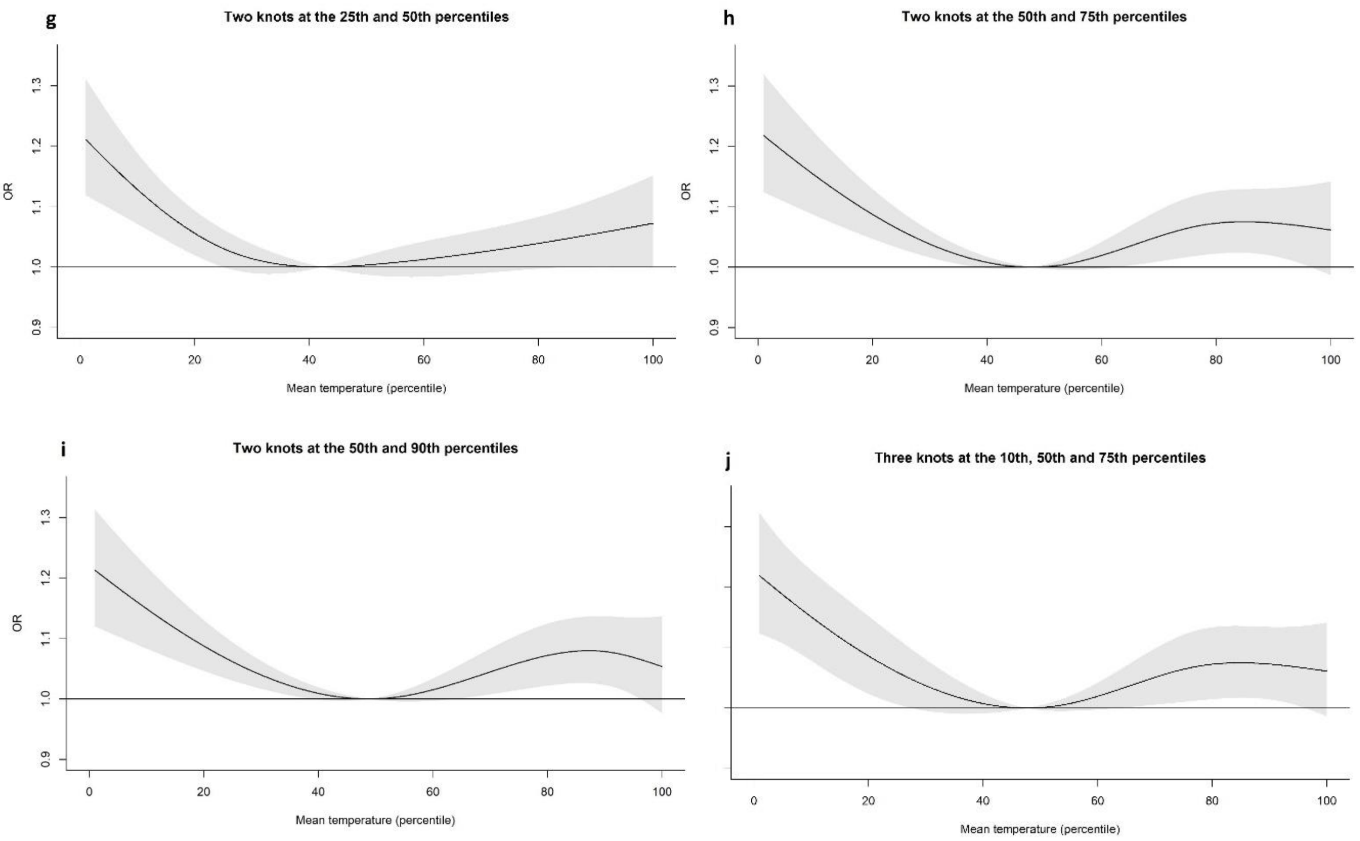
g
Two knots at the 25th and 50th percentiles
OR
Mean temperature (percentile)
h
Two knots at the 50th and 75th percentiles
OR
Mean temperature (percentile)
i
Two knots at the 50th and 90th percentiles
OR
Mean temperature (percentile)
j
Three knots at the 10th, 50th and 75th percentiles
Mean temperature (percentile)

Supplementary Fig. 6: Temperature-related neonatal deaths as a percentage of total neonatal deaths by country. a, Cold-related neonatal deaths as a percentage of total neonatal deaths in factual and counterfactual scenarios. **b,** Heat-related neonatal deaths as a percentage of total neonatal deaths in factual and counterfactual scenarios. **c,** Cold-related neonatal deaths attributable to climate change as a percentage of total neonatal deaths, with the corresponding 95% UI. **d,** Heat-related neonatal deaths attributable to climate change as a percentage of total neonatal deaths, with the corresponding 95% UI.

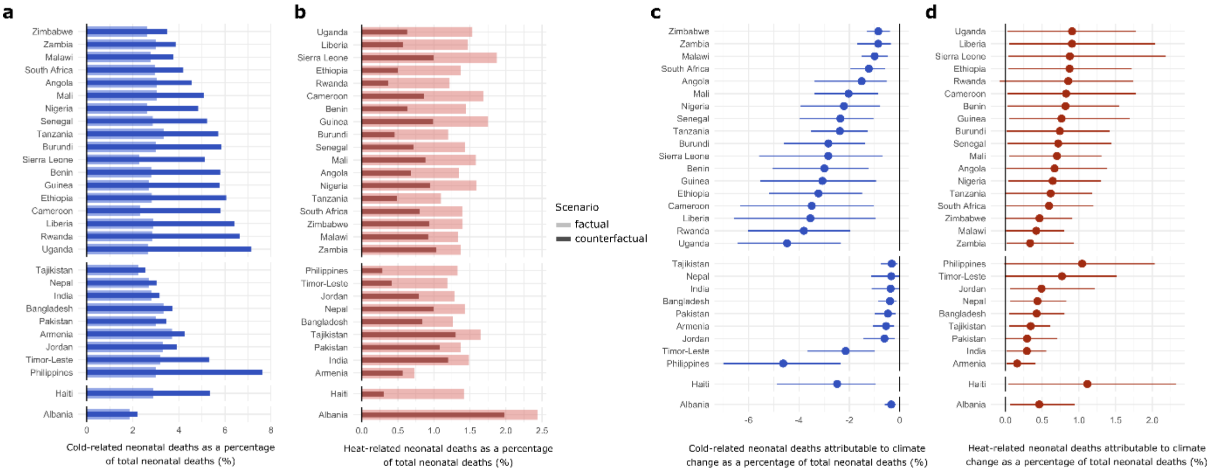

Supplementary Fig. 7: Temperature-related very early neonatal deaths as a percentage of total very early neonatal deaths by country. a, Cold-related very early neonatal deaths as a percentage of total very early neonatal deaths in factual and counterfactual scenarios. **b,** Heat-related very early neonatal deaths as a percentage of total very early neonatal deaths in factual and counterfactual scenarios. **c,** Cold-related very early neonatal deaths attributable to climate change as a percentage of total very early neonatal deaths, with the corresponding 95% UI. **d,** Heat-related very early neonatal deaths attributable to climate change as a percentage of total very early neonatal deaths, with the corresponding 95% UI.

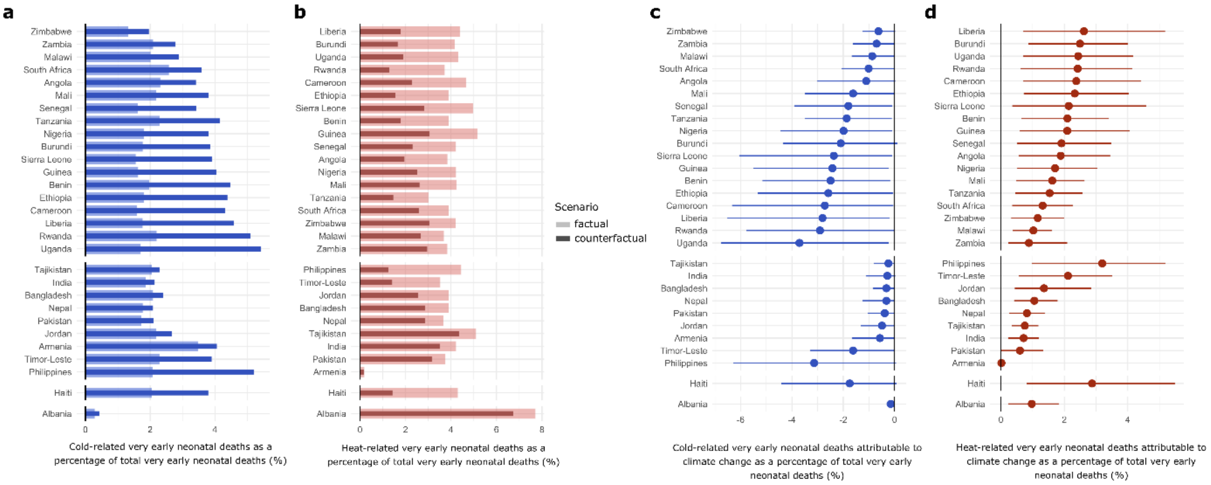

Supplementary Fig. 8: Flowchart of the included Demographic Health Surveys (DHS) and observations

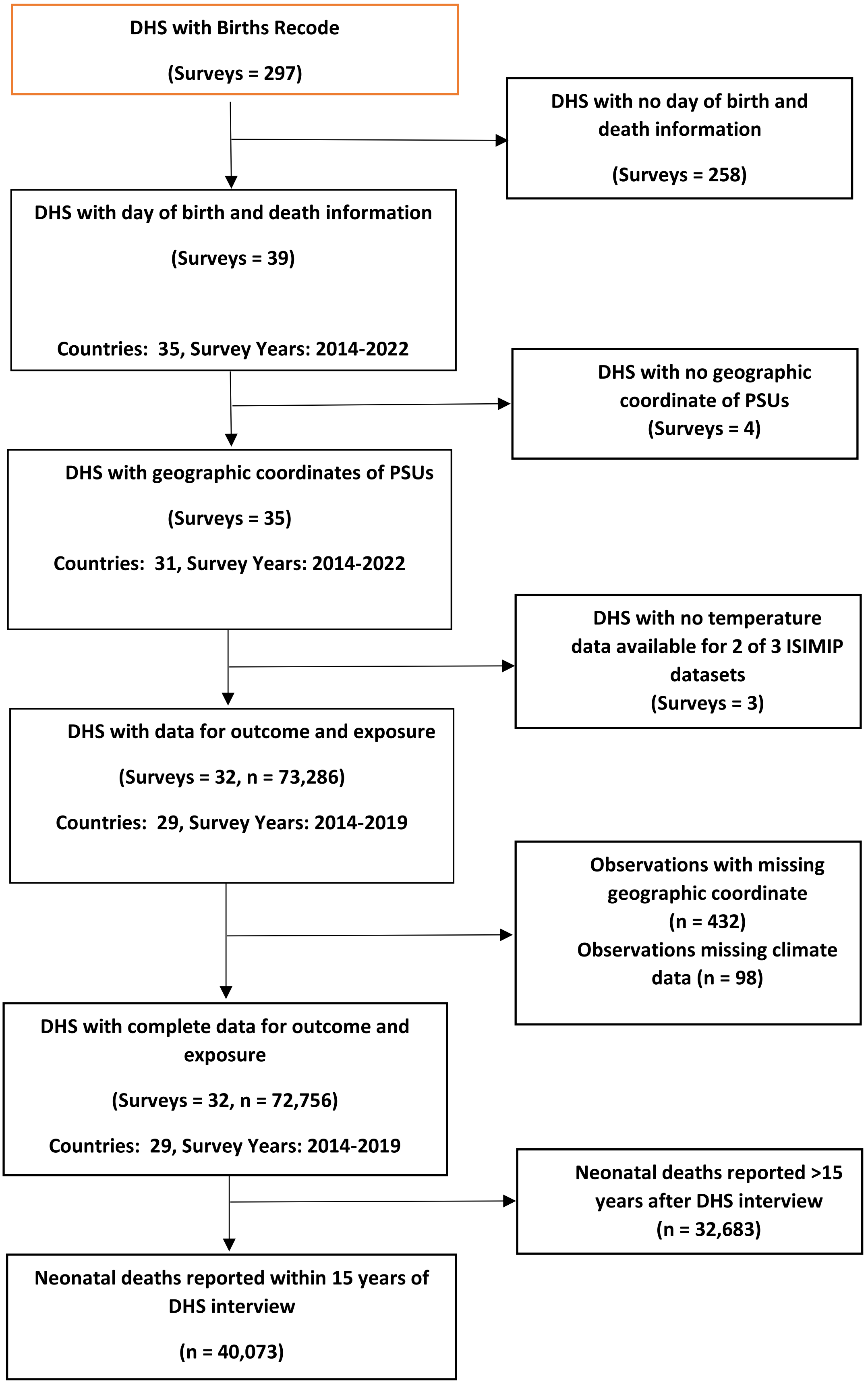

Supplementary Fig. 9: Plot of RR for neonatal mortality by temperature at specific lags (left) and RR by lag at 2.5th, 25.5th, 75.5th and 97.5th percentiles of temperature distribution (right), using temperature reanalysis dataset gswp3-w5e5.

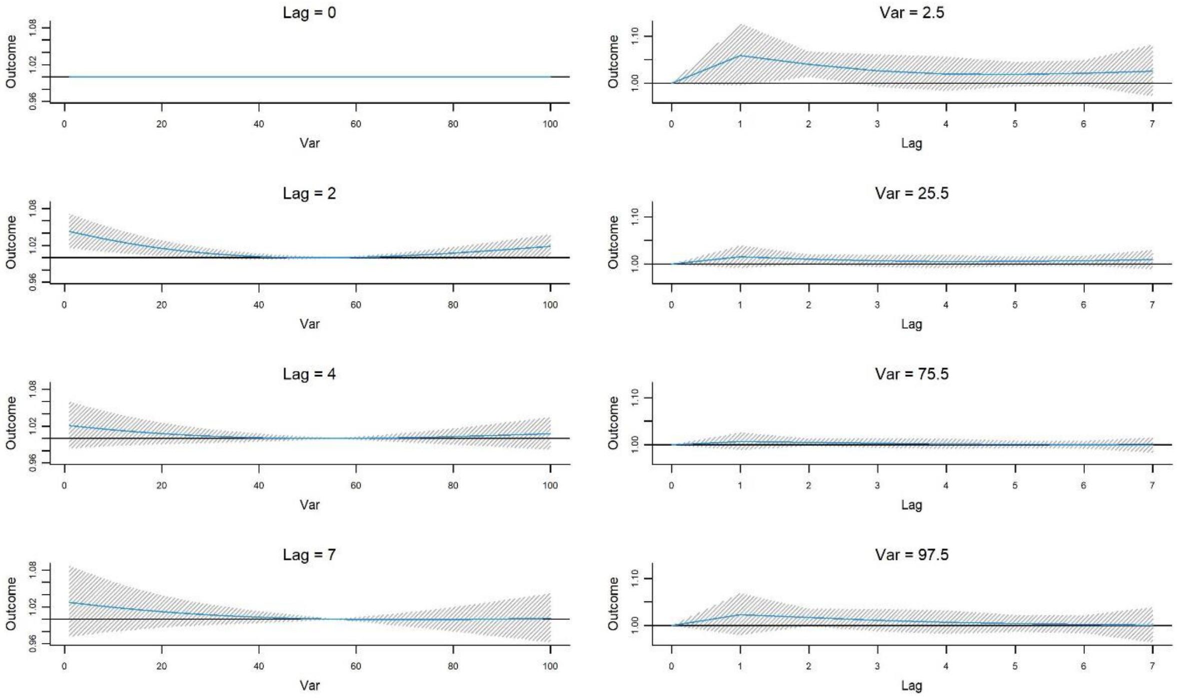

Supplementary Fig. 10: Plot of RR for very early neonatal mortality by temperature at specific lags (left) and RR by lag at 2.5th, 25.5th, 75.5th and 97.5th percentiles of temperature distribution (right), using temperature reanalysis dataset gswp3-w5e5.

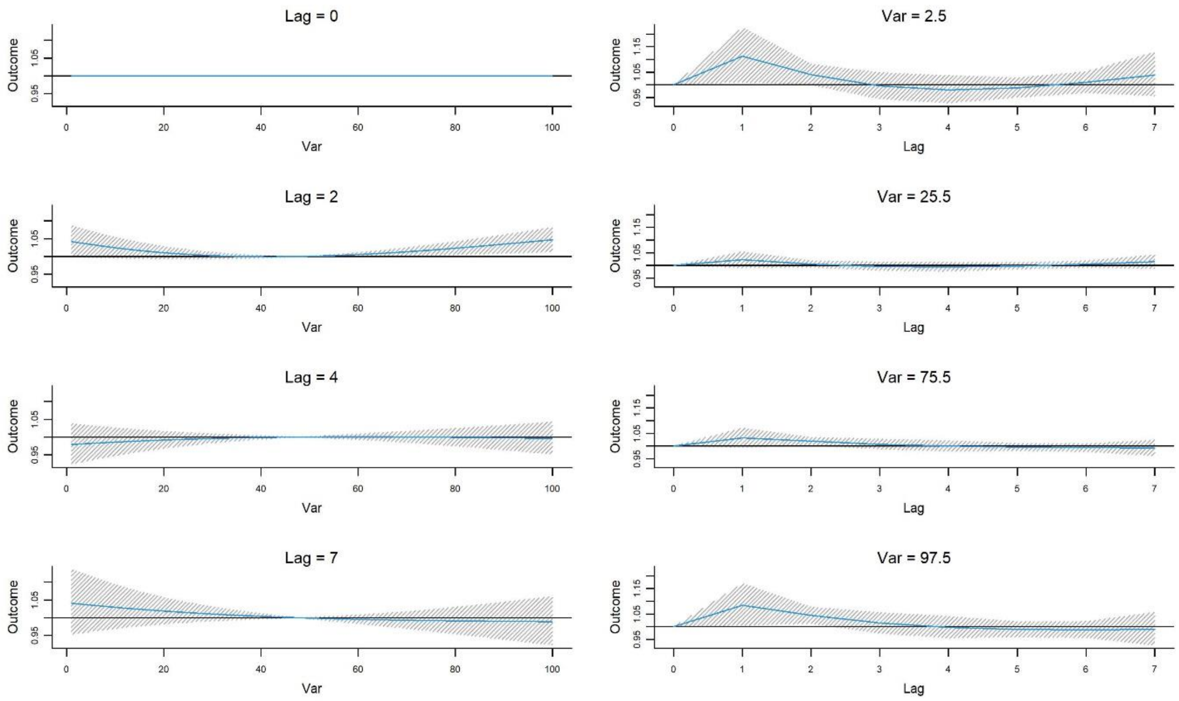

Supplementary Fig. 11: Temperature-related neonatal mortality attributable to climate change as a share of total neonatal mortality (%) across reanalysis products. a, Percentage of neonatal mortality attributable to climate change-induced reduction in cold. **b,** Percentage of neonatal mortality attributable to climate change-induced heat. Estimated as the difference in temperature-related neonatal mortality between the factual and counterfactual scenarios (2001-2019).

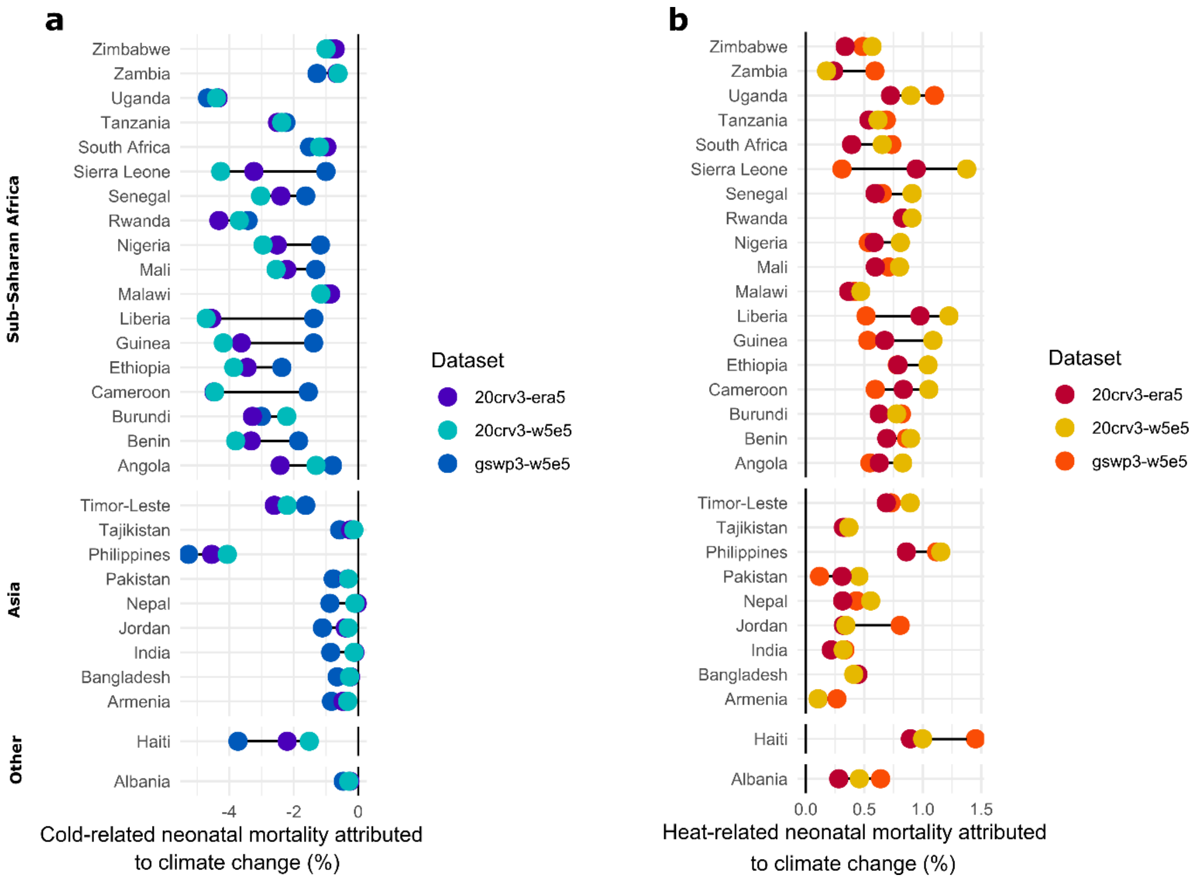

Supplementary Fig. 12: Temperature-related very early neonatal mortality attributable to climate change as a share of total very early neonatal mortality (%) across reanalysis products. a, Percentage of very early neonatal deaths attributable to climate change-induced reduction in cold. **b,** Percentage of very early neonatal deaths attributable to climate change-induced heat. Estimated as the difference in temperature-related very early neonatal mortality between the factual and counterfactual scenarios (2001-2019)

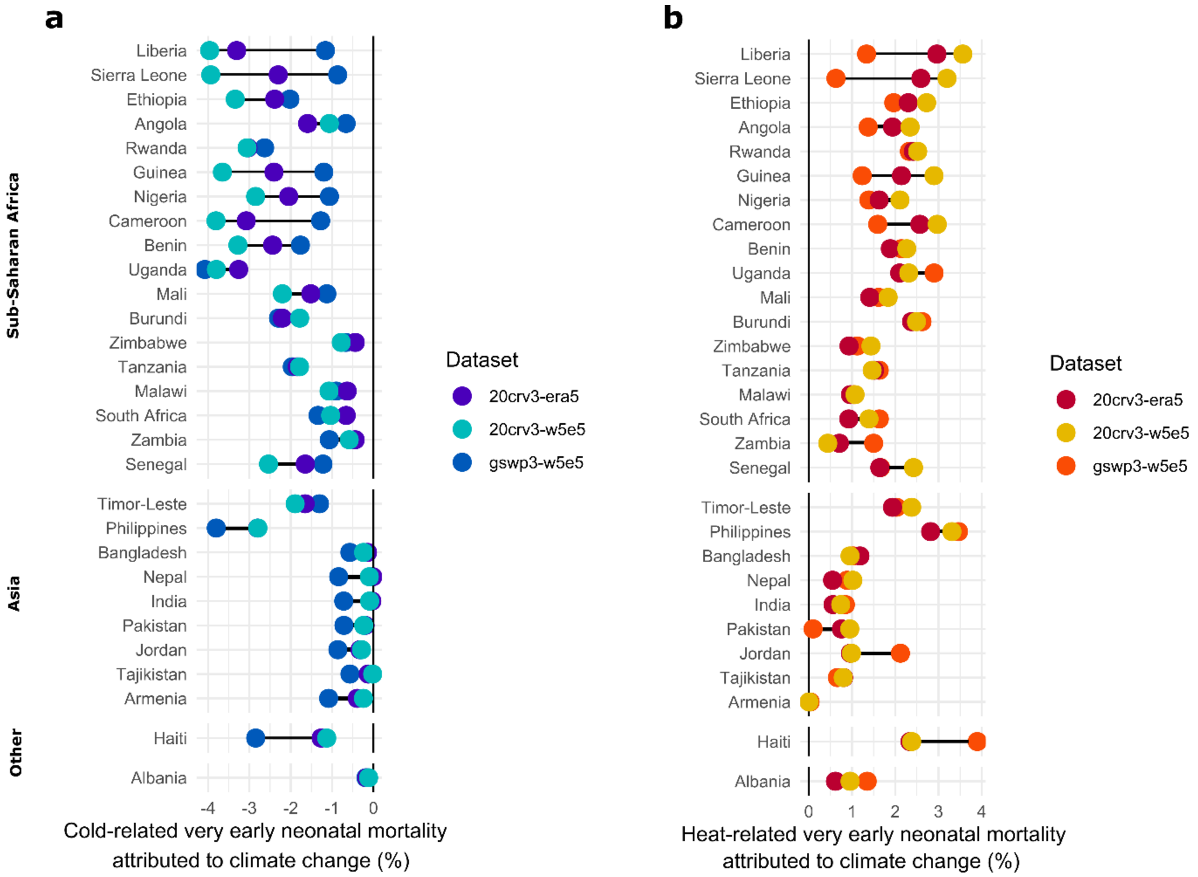

Supplement: Supplementary file 1 — Supplementary Information [file 41467_2024_49890_MOESM1_ESM.pdf]
